# The complete chloroplast genome sequence of *Potentilla bifurca* L.

**DOI:** 10.1080/23802359.2021.1923414

**Published:** 2022-05-02

**Authors:** Xiang Li, Yang Li

**Affiliations:** a School of Electronic and Information, Shaanxi Institute of Technology, Xi’an, China; b Xi’an Botanical Garden of Shaanxi Province, Institute of Botany of Shaanxi Province, Xi’an, China; c Shaanxi Engineering Research Centre for Conservation and Utilization of Botanical Resources, Xi’an, China

**Keywords:** *Potentilla bifurca*, chloroplast genome, phylogenetic analysis

## Abstract

*Potentilla bifurca* L. is a perennial herb in China, which has high ecological and economic values. Its complete chloroplast genome was reported in this study for the first time. The whole chloroplast genome was 157, 902 base pairs in length with 129 genes, including 84 protein-coding genes, 37 tRNAs, and 8 rRNAs. The maximum likelihood phylogenetic analysis revealed that the species of *P. bifurca* was isolated first among the genus *Potentilla*. This result will be helpful for the conservation and phylogeny programs of the genus *Potentilla*.

*Potentilla bifurca* L. is a perennial herb as a member of the *Potentilla* belonged to the family Rosaceae and widely distributed in China. As a creeping-rooted plant, *P. bifurca* plays an important role in the process of preventing steppe degradation, and usually becomes the dominant species in degraded ecosystems (Xu et al. 2019). Recent studies have shown that *P. bifurca* has high ecological and economic values on suppressing degradation, desertification and promoting vegetation restoration in arid and semi-arid grassland (Wang et al. 2014). Because *P. bifurca* has wide geographic distribution, and strong adaptability to stressful environments, it is important to understand how different evolutionary forces have sculpted the variation patterns in the genome of *P. bifurca*. In the present research, we characterized the whole chloroplast genome of *P. bifurca* and comprehended more about genetic information of this species, which can contribute to the understanding population genetics studies of *P. bifurca*.

For this study, *P. bifurca* were sampled from Duolun County (42°02′N, 116°17′E, 1324 m asl), Inner Mongolia Autonomous Region of China. A voucher specimen (HF2019003) was deposited at the Herbarium of Xi’an Botanical Garden of Shaanxi Province, China. DNA was extracted from the fresh leaves by modified CTAB method (Doyle and Doyle 1987). In order to construct the shotgun library, total genome DNA of *P. bifurca* was sequenced by Illumina Hiseq 2500 Sequencing System (Illumina, Hayward, CA). With *P. stolonifera* (NC_044418) as a reference, the qualified clean reads were assembled by NOVOPlasty (Dierckxsens et al. 2017). The complete chloroplast genome of *P. bifurca* was annotated by Geneious ver. 10.1 (http://www.geneious.com, Kearse et al. 2012) and online program Chloroplast Genome Annotation, Visualization, Analysis, and GenBank Submission (*CPGAVAS2*) (Shi et al. 2019). Finally, the validated complete chloroplast genome of *P. bifurca* was deposited in Genbank (Accession number MW255973).

The size of complete chloroplast genome of *P. bifurca* was 157, 902 bp in length, including a large single-copy region (LSC) of 86, 906 bp, a small single-copy region (SSC) of 18, 853 bp, and two inverted repeat (IR) regions of 26, 071 bp. The overall GC content is 37.0%. The genome contains 129 complete genes, including 84 protein-coding genes, 37 tRNA genes and 8 rRNA genes.

We used the complete chloroplast genomes sequence of *P. bifurca* and 8 other related species of *Potentilla* and two species: *Agrimonia pilosa* and *Sanguisorba officinalis* as out group from NCBI GenBank to construct phylogenetic tree. The 12 chloroplast genome sequences were aligned with MAFFT (Katoh and Standley 2013) and then the maximum likelihood (ML) tree was constructed by RAxML (Stamatakis 2014). The phylogenetic tree revealed that the species of *P. bifurca* was isolated first among the genus *Potentilla* with the support rate of 100% (Figure 1).

**Figure 1.**
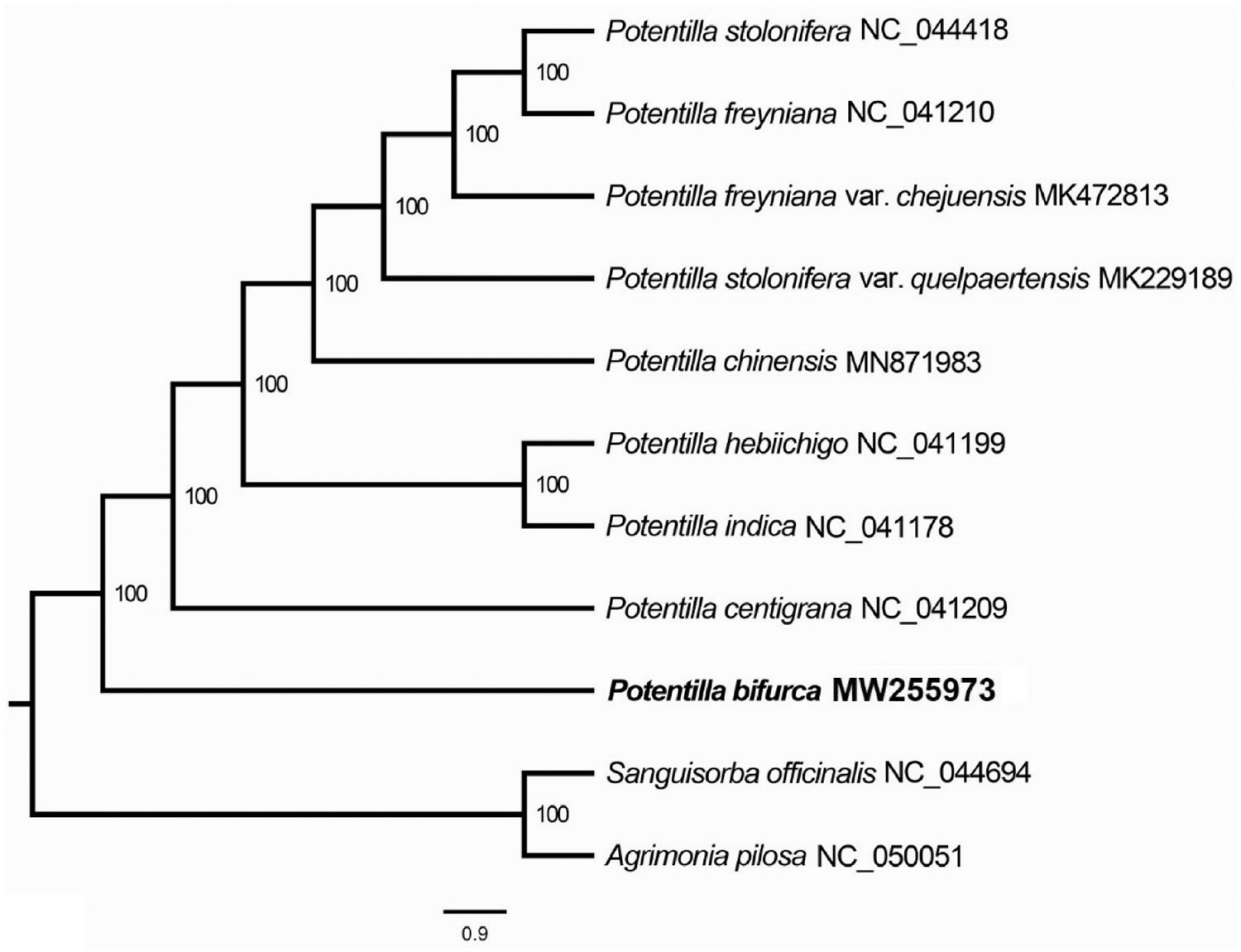
Maximum likelihood (ML) analysis of *P. bifurca* and other related species based on the complete chloroplast genome sequence.

## Data Availability

The genome sequence data that support the findings of this study are openly available in GenBank of NCBI at [https://www.ncbi.nlm.nih.gov] under the accession no. MW255973. The associated BioProject, SRA, and Bio-Sample numbers are PRJNA679129, SRR13077143, and SAMN16807717 respectively.
